# The risks of low hemoglobin deferral in a large retrospective cohort of plasmapheresis donors and the influence factors of return for a subsequent donation in China

**DOI:** 10.7717/peerj.14999

**Published:** 2023-03-13

**Authors:** Guanglin Xiao, Demei Dong, Ya Wang, Changqing Li, Gong tian Huang, Hui Yang, Jing Huang, Fei Chen

**Affiliations:** 1 Institute of Blood Transfusion, Chinese Academy of Medical Sciences and Peking Union Medical College, Chengdu, China; 2 Beijing Tiantan Biological Products Company Limited, Beijing, China; 3 Shanghai RAAS Blood Products Company Limited, Shanghai, China; 4 Nanyue Biopharming Corporation Ltd, Hengyang, China; 5 Jiange Plasmapheresis Station, Sichuan Yuanda Shuyang Pharmaceutical Company Limited, Guangyuan, China; 6 Cangxi Plasmapheresis Station, Sichuan Yuanda Shuyang Pharmaceutical Company Limited, Guangyuan, China

**Keywords:** Plasmapheresis donors, Hemoglobin, Low hemoglobin deferral, Safety, Recruitment

## Abstract

**Background:**

According to the Technical Operation Procedures for Plasmapheresis Collection Station (2019) in China, plasmapheresis donors with low hemoglobin (Hb) levels (men <12.0 g/dL; women <11.0 g/dL) were deferred for at least 2 weeks. The purpose of this retrospective study was to survey the demographic characteristics of plasmapheresis donors with low Hb deferral (LHD) and identify at-risk LHD donors, so as to enhance donor safety and improve donation service management.

**Methods:**

From 2018 to 2020, a multi-center study involving plasmapheresis donors from 18 plasmapheresis centers in three provinces (Sichuan, Yunnan and Hunan) of China was conducted. Donor demographics (age, sex) and donation information (date of donation, first-time donors *vs*. repeat donors, the number of lifetime donations, the number of donations in the last 12 months, and whether the LHD donor returned for a subsequent donation) were collected. The Cochran-Mantel-Haenszel method was used to explore the risk factors for LHD while adjusting for the different provinces. Logistic regression analysis was used to investigate the factors influencing the return for a subsequent donation after LHD.

**Results:**

A total of 497,039 plasmapheresis donors were included. Female donors’ LHD rate was 0.15% on average, while male donors’ LHD rate was 0.01%. Female donors aged 41–50 years old (OR: 2.276, 95% CI [1.333–3.887], *p* = 0.002) were more likely to experience LHD temporarily than those aged 18–30 years old. For female donors, compared with donations in the winter, they had a higher risk for LHD in the summer (OR: 2.217, 95% CI [1.670–2.943], *p* < 0.001), spring (OR: 2.402, 95% CI [1.806–3.196], *p* < 0.001), and fall (OR: 2.002, 95% CI [1.500–2.673], *p* < 0.001). Among the LHD donors, those who had donated more frequently in the past were more likely to return for a subsequent donation (*p* = 0.012).

**Conclusions:**

Female donors were at a higher risk of LHD, particularly between the ages of 41 and 50. A clear seasonal pattern in the rate of LHD was observed. In the winter, the risk of LHD was the lowest; thus, it was advised to recruit plasmapheresis donors throughout the winter and to make the required adjustments for recruitment measures during other seasons. The number of previous donations was correlated with the return rate after LHD. Our observations could have practical implications for plasmapheresis donor management.

## Introduction

Plasmapheresis donation provides the starting material for the further manufacture of plasma-derived medical products such as albumin, immunoglobulin, and clotting factors (Schreiber et al., 2018). Currently, China is one of the largest consumers in the global plasma-derived products market. This regional market is growing at a rapid pace with the increasing demands of a huge population of more than 1.4 billion (Wang et al., 2018). While there were only 3.67 million registered plasmapheresis donors in China by 2019 (National Health Commission of the PRC, 2019b). China’s blood product supply is insufficient due to the country’s vast population and rising demand. For instance, 60% of the Chinese market is made up of imported albumin. A significant portion—nearly 37%—of the albumin produced in the United States was sold to China (CHYXX, 2020). Reaching national self-sufficiency for plasma products in China has been significantly hampered by the lack of source plasma for fractionation. Keeping as many fixed plasmapheresis donors as possible is a significant and practical solution to mitigate plasma scarcity.

Donor deferral was well documented to have a detrimental effect on both first-time and repeat donors’ likelihood of returning to donate (Halperin, Baetens & Newman, 1998; Masser et al., 2008). Low hemoglobin (Hb) (men <12.0 g/dL; women <11.0 g/dL) was the frequent reason for deferral of plasmapheresis donation, which accounted for 14.1% of all the reasons (other reasons *e.g*., blood pressure/pulse unacceptable, temperature unacceptable) for delayed donation in 2017–2018 in the United States (Schmidt et al., 2022). Plasmapheresis donors who had been deferred due to low Hb had a considerably lower likelihood of returning for plasmapheresis donation in the future. According to estimates, donors who were deferred for low Hb provided roughly 30% fewer donations over the course of the next 4 to 5 years than those whom were not deferred (Halperin, Baetens & Newman, 1998).

Because whole blood donation would cause the loss of red blood cells (RBCs), some articles have surveyed the low Hb deferral (LHD) of whole blood donors (Baart et al., 2012; Cable et al., 2012; Pasricha et al., 2011). While little attention has been given to plasmapheresis donors. Plasmapheresis donors lost approximately 30 mL of whole blood per donation, including a volume taken for laboratory testing, hemolysis due to the plasmapheresis procedure, and the small volumes remaining in the plastic collection systems. If a plasmapheresis donor made 24 donations a year, 720 mL of whole blood would be lost. The cumulative effect of a minor blood loss at each donation still increased the risk of low Hb. In general, it is also important to survey the LHD in plasmapheresis donors.

Chinese plasmapheresis donation standards differ from others. In China, the interval between plasmapheresis donations is 14 days (National Health Commission of the PRC, 2019a), while US donors may donate twice weekly (FDA, 2003). Chinese plasmapheresis donors can not contribute with more than 580 g of plasma (without anticoagulant) for each donation (National Health Commission of the PRC, 2019a), while US donors can reach 625–800 ml (without anticoagulant) (FDA, 2003). Previous foreign studies cannot be extrapolated to Chinese plasmapheresis donors due to differences in collection standards, ethnicity, and eating habits. It is essential to understand the risks of LHD in Chinese plasmapheresis donors and develop effective strategies to keep these deferred plasmapheresis donors motivated to return and donate.

## Materials and Methods

### Study population and data collection

A total of 497,039 plasmapheresis donors from three provinces (Sichuan, Yunnan and Hunan) of China were included in our cross-sectional study population. The inclusion interval for this study was from 2018 to 2020. This study was approved by the Ethics Committee of the Institute of Blood Transfusion, Chinese Academy of Medical Sciences (IBT) (NO: 2021042).

Donor selection in China included donor health assessment and blood detection. Donor assessments should include health consultations and physical exams by trained assessors. Plasmapheresis donors must answer health and lifestyle questions truthfully. The donor physical examination and consultation questionnaire were based on the technical operation procedures for plasmapheresis collection station (2019) in China (National Health Commission of the PRC, 2019a). The donor questionnaire currently included medical history, including fever, dental work, diarrhea, infections, malaria, typhoid, prescribed aspirin, vaccination, allergies, ulcers, diabetes, convulsions or fainting spells, cancer, surgery, history of blood transfusion, menstrual period and pregnancy in females, risk behaviors like alcoholism, prostitution, illicit drug use, sex with prostitutes, male homosexuality, and having a sexual part. Before donating, donor blood samples must be tested for Hb, alanine aminotransferase (ALT), hepatitis B, and syphilis.

Deferred donors were those who failed the physical exam or blood test (plasmapheresis donor selection criteria in China were shown in Table 1). Temporary deferral caused include being on medication, occasional abnormal blood pressure or pulse, raised ALT, low Hb level. Permanent deferral was caused by serious illnesses, high-risk behaviors, and “reactive” hepatitis B or syphilis tests.

**Table 1:** Plasmapheresis donor selection criteria in China.

Parameters	Values
Age	18–60 years
Weight	≥50 Kg for males and ≥45 Kg for females
Blood pressure	Systolic between 90 and 140 mm Hg *vs*. diastolic between 60 and 90 mm Hg *vs*. pulse pressure difference ≥30 mm Hg
Pulse	Regular pulse between 60–100 beats per minute
Temperature	Normal
Hb	≥120 g/L for males and ≥110 g/L for females
Serum/plasma protein	Serum protein ≥60 g/L; plasma protein ≥50 g/L
Alanine aminotransferase (ALT)	≦50 μ/L for both males and females
Hepatitis B virus surface antigen (HBsAg)	Negative
Hepatitis C virus antibody (HCVAb)	Negative
HIV-1 and HIV-2 antibody	Negative
Syphilis	Negative
Donation interval	2 weeks
Serum/plasma electrophoresis	Compared with the normal plasma electrophoretogram, the electrophoretogram bands of donors did not increase or decrease. Albumin content not less than 50%.
Temporary deferral	Prospective plasmapheresis donor ineligible in donation for a time-limited period and can return for further donation
Permanent deferral	Prospective plasmapheresis donor unable to donate forever for one or a variety of reasons

The result of interest was the incidence of a deferred visit because of low Hb. Donor deferral because of reasons other than a low Hb level was eliminated. Donor data was retrieved by the Donor Management System from each plasmapheresis center. Donation information included the date of donation, total number of lifetime donations and number of donations in the last 12 months. Demographic factors included sex and age. According to previous studies, seasonal temperature variation was related to the rate of donor deferral for low hematocrit (HCT) (Sebok et al., 2007). We divided the date of donation into four groups according to seasons: winter (December 1-February 29), spring (March 1-May 31), summer (June 1-August 31), and fall (September 1-November 30).

### Hb testing

Before each donation, Hb testing was performed on venous blood samples. The copper sulfate technique (CST), which was a qualitative method for measuring Hb based on the estimate of specific gravity from a blood sample. LHD was recorded as present if the gravity value was below the cut-off value: 1.052 for men and 1.050 for women at 20 °C, which correspond to a Hb concentration of 120 and 110 g/L, respectively (National Health Commission of the PRC, 2019a). Some centers used the azide methemoglobin method to estimate Hb (Vanzetti, 1966). A domestic study (Dai et al., 2015) verified that there were no significant differences in specificity, accuracy or precision among the CST and the azide methemoglobin method according to ICHQ2a and ICHQ2R1 methodology verification requirements (European Medicines Agency, 1995; FDA, 2015; ICH, 2005; Walfish, 2015).

### Statistical analyses

All statistical analyses were performed for men and women separately, and therefore the difference in sex distribution could not influence the results. Percentages were used to express categorical variables. We separated the population into categories based on sex, age, donation year and seasons. The Cochran-Mantel-Haenszel Method was used to explore the risk variables of LHD adjusting for the different provinces. *P*-values were adjusted for multiplicity of testing using the Bonferroni method. To quantify the strength of the link, an odds ratio and a 95% confidence interval were calculated.

We also investigated the characteristics that influence the return for a subsequent donation in LHD plasmapheresis donors. In this study, univariate logistic regression was used, and then the independent variables would be entered into a multivariate logistic regression model. The independent variables included donor sex, age, total number of lifetime donations and number of donations in the last 12 months. If no special statements, the criterion for the *p* value to be considered significant was at the 5% level. Statistical analyses were conducted with the Statistical Product and Service Solutions (SPSS) application version 24. Graphical analyses were performed using the statistical software (R 4.1.2; R Core Team, 2021), and the forestplot package was used.

## Results

### Baseline characteristics

**Figure 1:**
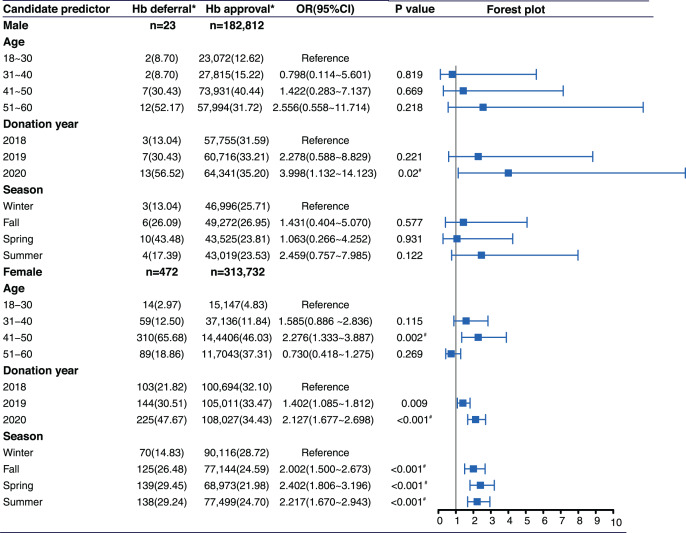
Distribution of candidate predictors and Hb deferral for males and females separately. An asterisk (*) indicates that the Cochran-Mantel-Haenszel test, which controlled for statistical differences between provinces, revealed a significant difference between the investigation group and the reference group after Bonferroni correction (Donation Year group: *p* < 0.05/2 = 0.025; Age and Season group: *p* < 0.05/3 = 0.0167). CI, confidence interval; OR, odds ratio.

During the study period from 2018 to 2020, 18 plasmapheresis centers registered a total of 497,039 plasmapheresis donors. The characteristics of all plasmapheresis donors were listed in Table 2. The data showed that most plasmapheresis donors were women (*n* = 314,204, 63.22%), aged 41–50 years old (46.06%). While 36.78% (*n* = 182,835) of the donors were men, with the majority being between the age of 41 and 50 (40.44%). The number of plasmapheresis donors increased with the year of study both in male and female donors. Only 495 plasmapheresis donors (23 male and 472 female donors) were temporarily deferred because of low Hb (Fig. 1). The overall prevalence of LHD was 0.10% (495/497,039), and the sex-standardized prevalence rate of LHD was 0.11%.

**Table 2:** Demographic information of plasmapheresis donors.

Demographic information	Number of donations*	
Total	497,039	
Gender		
Male	182,835 (36.78)	
Female	314,204 (63.22)	
Age	Male	Female
18–30	23,074 (12.62)	15,161 (4.83)
31–40	27,817 (15.21)	37,195 (11.84)
41–50	73,938 (40.44)	144,716 (46.06)
51–60	58,006 (31.73)	117,132 (37.28)
Donation year	Male	Female
2018	57,758 (31.59)	100,797 (32.08)
2019	60,723 (33.21)	105,155 (33.47)
2020	64,354 (35.20)	108,252 (34.45)
Season	Male	Female
Spring	43,535 (23.81)	69,112 (22.00)
Summer	43,023 (23.53)	77,637 (24.71)
Fall	49,278 (26.95)	77,269 (24.59)
Winter	46,999 (25.71)	90,186 (28.70)

**Note:**

* Data are reported as *n* (%).

### Incidence of LHD and analysis of risk factors

#### Sex

Sex was the predominant factor of LHD. There were 23 male and 472 female donors who were deferred because of low Hb level. The prevalence of LHD in female donors was 0.15% (472/313,732). The prevalence of LHD in male donors was 0.01% (23/182,812) (Fig. 1). Female donors were deferred at a significantly higher rate than male donors (*p* < 0.001). The standardized prevalence of LHD for female and male donors were unchanged after adjustment for age.

#### Age

**Figure 2:**
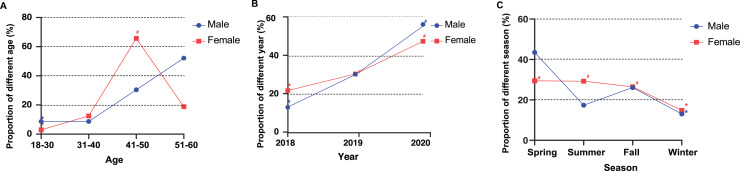
The association between different characteristics and the risk of Hb deferral in male and female plasmapheresis donors. An asterisk (*) indicates the reference group. A number sign (#) indicates that a significant difference was found between the investigation group and reference group after Bonferroni correction. (A) Proportion of different age: *p* < 0.05/2 = 0.025; (B) proportion of different years: *p* < 0.05/3 = 0.0167; (C) proportion of different seasons: *p* < 0.05/3 = 0.0167.

In male donors, there was no significant correlation between age and the risk of LHD. In female donors, age 41–50 years old (OR: 2.276, 95% CI [1.333–3.887], *p* = 0.002) were more likely to be deferred than those aged 18–30 years old (Figs. 1 and 2A).

#### Donation year

Male donors who donated in 2020 (OR: 3.998, 95% CI [1.132–14.123], *p* = 0.02) were more likely to be deferred than those who donated in 2018. Compared to donations in 2018, female donors who donated in 2020 (OR: 2.127, 95% CI [1.677–2.698], *p* < 0.001) and in 2019 (OR: 1.402, 95% CI [1.085–1.812], *p* = 0.009) were more likely to experience LHD (Fig. 1). The relationship between donation year and risk of LHD was linear in both male and female donors (Fig. 2B).

#### Season

Seasons had no statistically significant association with LHD in male donors. In female donors, compared with donations in the winter, they had a higher risk for LHD in the summer (OR: 2.217, 95% CI [1.670–2.943], *p* < 0.001), spring (OR: 2.402, 95% CI [1.806–3.196], *p* < 0.001), and fall (OR: 2.002, 95% CI [1.500–2.673], *p* < 0.001) (Figs. 1 and 2C).

### Influence factors of return for a subsequent donation

Of all the LHD donors (*n* = 495), a total of 136 (27.47%) donors returned and re-donated successfully (seven males and 129 females), and 359 (72.53%) donors have not returned for donation. Results from the univariate analysis showed that repeat donors were more likely to return to donation (*p* = 0.025) compared with first-time donors. The repeat donors were further divided into groups according to the total number of lifetime donations and the number of donations they had made in the last 12 months. With the increase of the total number of donations (*p* = 0.003) and number of donations in last 12 months (*p* < 0.001), the rate of re-donation increased (Table 3).

**Table 3:** Summary of reactivated donors among all LHD donors and univariate logistic regression.

Factors		Number of donors	Re-donation*	No re-donation*	*p* value
Total number		495	136 (27.47)	359 (72.53)	
Gender					0.745
Male		23	7 (30.43)	16 (69.57)	
Female		472	129 (27.33)	343 (72.67)	
Age					0.917
18–30		16	4 (25.00)	12 (75.00)	
31–40		61	19 (31.15)	42 (68.85)	
41–50		317	86 (27.13)	231 (72.87)	
51–60		101	27 (26.73)	74 (73.27)	
Donor type					0.025
First-time donor		106	20 (18.87)	86 (81.13)	
Repeat donor		389	116 (29.82)	273 (70.18)	
Total number of donations					0.003
0		106	20 (18.87)	86 (81.13)	
1–50		292	75 (25.68)	217 (74.32)	
51–100		61	24 (39.34)	37 (60.66)	
101–150		25	11 (44.00)	14 (56.00)	
>150		11	6 (54.55)	5 (45.45)	
Number of donations in last 12 months					<0.001
0		219	40 (18.26)	179 (81.74)	
1–10		145	42 (28.97)	103 (71.03)	
11–20		69	24 (34.78)	45 (65.22)	
>20		62	30 (48.39)	32 (51.61)	

**Note:**

* Data are reported as *n* (%).

**Figure 3:**
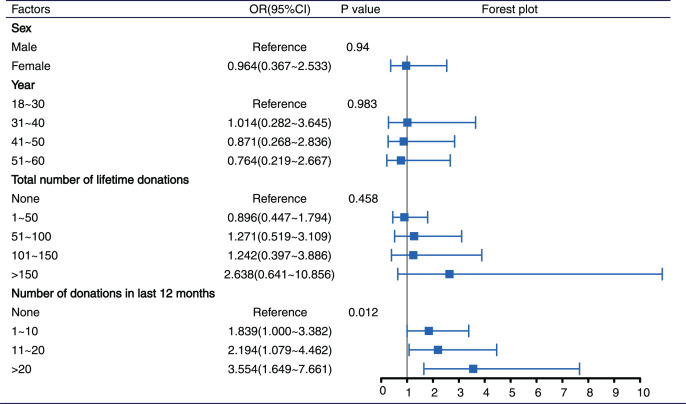
Influence factors of return for a subsequent donation by using multivariate logistic regression. CI, confidence interval; OR, odds ratio.

All variables were included in multivariate logistic analysis (enter method). Donor type (first-time and repeat donors) was not simultaneously entered into models due to collinearity. After adjustment for other confounders, compared to no donation in the last 12 months, the return ratios for 1 to 10, 11 to 20, and >20 donations in the last 12 months were 1.839 (95% CI [1.000–3.382]), 2.194 (95% CI [1.079–4.462]), and 3.554 (95% CI [1.649–7.661]), respectively. The return ratios increased with the number of donations in the last 12 months (*p* = 0.012). The number of previous donations was correlated with return rates after LHD. Other characteristics, such as sex, year of donation, and total number of lifetime donations, had a minimal impact on the return ratios (Fig. 3).

## Discussion

This study examined the deferral rate for low Hb level among plasmapheresis donors in China. The overall prevalence of LHD was 0.10% in 2018–2020, which was lower than that in the United States. In a more recent survey of 255 plasmapheresis sites in the United States, the plasmapheresis donors’ LHD rate was 0.1% in 2017–2018 and 1.7% in 2018–2020 (Schmidt et al., 2022). Most likely, the disparity in LHD rates between China and the United States was attributable to the frequency of donation. In the United States, a donor could donate plasma twice in 7 days with at least 48 h between each donation (FDA, 2003); in China, the interval between plasmapheresis donations was 2 weeks (National Health Commission of the PRC, 2019a). A shorter donation interval in the United States was not conducive to the Hb recovery of some donors. We should also note that the FDA set the minimum donor Hb criteria at 13.0 g/dL for men and 12.5 g/dL for women (Association for the Advancement of Blood & Biotherapies (AABB), 2015), while in China, men and women had minimum Hb requirements of 12.0 and 11.0 g/dL, respectively. Lower minimal Hb criteria for donors in China may be a significant factor in the lower LHD rate. The LHD rate of plasmapheresis donation in this study was also lower than that of other forms of donation. It was lower than that reported for whole blood donation: 5% in the Netherlands (Baart et al., 2012); 10% in the United States (Newman, 2008); 1.9% in Colombia (Urbina et al., 2021). It was also lower than platelet donations: 9.8% in the United States (Mast et al., 2010) and 9.1% in Columbia (Urbina et al., 2021). These various LHD rates among donations could be a result of the various products and procedures.

In this study, women had a 15-fold higher LHD risk than men. LHD rates differed between men and women, as previously reported (Halperin, Baetens & Newman, 1998). According to data from 715,000 whole blood donors, female was independently associated with LHD (Mast et al., 2010). Some studies revealed that female donors had a 14-fold higher risk of LHD compared with male donors (OR 14.62, 95% CI [12.43–17.19]) (Browne et al., 2020; Bullock et al., 2010). Due to their monthly blood loss, premenopausal women were prone to low Hb. Other causes of higher Hb in men included elevated testosterone levels in men, which resulted in elevated Hb (Marks et al., 2014), as well as the Hb increase associated with cigarette smoking (Prados Madrona et al., 2014).

For male donors, our study found no association between age and LHD risk. LHD risk increased with age in male whole blood donors and platelet donors in the United States (Mast et al., 2010). A study conducted in the Netherlands also indicated that the risk of LHD rose with age in male whole blood donors (Baart et al., 2012). In our study, a small sample of male donors with LHD might explain some of these disparities. The results from this study showed that LHD risk increased in women aged 18–50 but decreased after aged 50. The drop in LHD risk after aged 50 could be attributed to the menopause, as women no longer lose Hb through menstruation.

The study found surprising LHD prevalence in different donation years. We saw that the LHD rate rose over time. A US study found that plasmapheresis donors’ unsatisfactory HCT deferral ratio rose from 0.1% in 2017–2018 to 1.7% in 2019–2020 (Schmidt et al., 2022). They thought the increasing plasma collections might cause the rise. This might also explain the increasing LHD rate among Chinese plasmapheresis donors: in 2018, the industry collected more than 8,600 tons of plasma, up nearly 7% from the year before; in 2019, it collected 9,200 tons, up 6% (CHYXX, 2020). Although the total number of plasmapheresis donors had increased, it did not rule out that some plasmapheresis donors had switched from low-density to high-density donation.

We found a seasonal pattern in LHD in a large plasmapheresis donor cohort, notably female donors. Compared to winter, LHD rates were twice as high in other seasons. The LHD rates in spring, summer, and fall were similar. Hb was mostly affected by temperature. Our findings matched past research (Sebok et al., 2007; Borgna-Pignatti et al., 2006; Lau, Hansen & Sererat, 1988; Thirup, 2003). They demonstrated that the seasonal impact of the HCT deferral rate was greatest in regions of the United States with the greatest temperature variation. In a cross-sectional study, 82 healthy adult men’ HCT decreased (−2%) throughout the summer (Kolar et al., 1988). Trends may have several causes. Hemodilution, which reduced blood viscosity by transferring water to the vascular system, was the most likely cause of seasonal Hb variations (Bass et al., 1955; Senay & Kok, 1976; Shapiro et al., 1981; Wyndham et al., 1968). Seasonal changes in nutrition, exercise, and viral illnesses might also impact Hb levels (such as parvovirus). Our research lacked such information, so we couldn’t draw any conclusions. The method, CST, used to monitor Hb levels may have been slightly affected by environmental temperature changes. During the summer, plasmapheresis centers have to put in extra effort to maintain a sufficient plasma supply. We recommended inviting donors especially during the winter months to increase the chances of a successful plasmapheresis donation.

Many donors did not return to donation due to LHD, even if they had fulfilled the Hb requirements. Some studies showed that after a temporary delay, most first-time and repeat donors didn’t donate again (Halperin, Baetens & Newman, 1998; Lawson-Ayayi & Salmi, 1999; Linden, Gregorio & Kalish, 1988). Understanding which characteristics had the most impact on re-donations would help target LHD donors with the proper donor education and recruitment tools. In our study, we found a considerable positive correlation between the number of donations in the last 12 months and subsequent donations. The higher the frequency of recent donations, the greater the possibility of meeting Hb requirements again and continuing to donate. Plasmapheresis centers should create appropriate reentry procedures for this population. At the same time, it was suggested to increase efforts for the recruitment of LHD donors who had donated less in the past 12 months, especially first-time donors, and encouraged them to continue to donate.

Nonetheless, this study has numerous potential shortcomings. First, the low Hb cutoff chosen was lower than what the WHO (Association for the Advancement of Blood & Biotherapies (AABB), 2015) and AABB (World Health Organization (WHO), 2012) suggested. Therefore, the percentage of LHD could not be compared to that of other countries. Second, the Hb detection method, CST, did not achieve a satisfactory level of accuracy (Tondon et al., 2009). It was widely used in China until 2020 because it was a low-cost and simple method. Since 2021, it has been replaced by non-invasive hemoglobin and blood cell analyzers. Third, there were other diseases that can cause low Hb, such as alpha-thalassemia, but we cannot obtain such data from the Donor Management System. Fourth, we could not strictly control the environmental temperature for Hb detection, which may affect the accuracy of detection. Finally, in this study, the donation history of non-LHD donors was unavailable, so we were unable to compare and investigate the impact of the frequency and total number of plasmapheresis donations on LHD, which would be the subject of future research.

## Conclusion

Low Hb is a frequent reason for plasmapheresis donor deferral. In this study, we attempted to better comprehend the factors that influence LHD and subsequent donations. In general, the LHD ratio of plasmapheresis donors in China was lower than in the earlier study conducted in the United States. This was likely related to the varying donation intervals. The results still indicated that premenopausal women should be targeted. Inviting plasmapheresis donors during the winter may increase the likelihood of a successful donation. The frequency of donations made in the last 12 months was significantly correlated with return rates after LHD. These data provided useful information that might be used to identify high-risk groups with LHD, identify groups with the potential to return, and drive policy decisions.

## Supplemental Information

10.7717/peerj.14999/supp-1 Supplemental Information 1 Basic characteristics of LHD donors.

10.7717/peerj.14999/supp-2 Supplemental Information 2 Summary of plasmapheresis donation information from 18 plasmapheresis stations in 2018-2020.

## Additional Information and Declarations

### Data Availability

The following information was supplied regarding data availability: The raw data is available in the Supplemental Files.
